# Tetra­kis[μ-1,3-bis­(4,5-dihydro-1,3-oxazol-2-yl)benzene-κ^2^ *N*:*N*′]tris­ilver(I) tris­(hexa­fluoridophosphate)

**DOI:** 10.1107/S1600536812034721

**Published:** 2012-08-11

**Authors:** Chun-Wei Yeh, Yuh-Wen Ho, Hsun-Tsing Lee, Ju-Chun Wang, Maw-Cherng Suen

**Affiliations:** a Department of Chemistry, Chung-Yuan Christian University, Jhongli 32023, Taiwan; b Department of Creative Fashion Design, Taoyuan Innovation Institute of Technology, Jhongli 32091, Taiwan; c Department of Materials Science and Engineering, Vanung University, Jhongli 32061, Taiwan; d Department of Chemistry, Soochow University, Taipei, Taiwan

## Abstract

In the title compound, [Ag_3_(C_12_H_12_N_2_O_2_)_4_](PF_6_)_3_, one Ag^I^ ion, lying on a twofold rotation axis, is coordinated by four N atoms from four 1,3-bis­(4,5-dihydro-1,3-oxazol-2-yl)benzene (*L*) ligands in a distorted tetra­hedral geometry and the other Ag^I^ ion is coordinated by two N atoms from two *L* ligands in a bent arrangement [N—Ag—N = 169.03 (17)°]. Two *L* ligands adopt a *syn* conformation, while the other two adopt an *anti* conformation. They bridge adjacent Ag^I^ ions, forming a trinuclear complex. One of the PF_6_^−^ anions is half-occupied, with the P atom located on a twofold rotation axis. The PF_6_^−^ anions link the complex mol­ecules *via* Ag⋯F inter­actions [2.80 (2) and 2.85 (2) Å] into a polymeric chain along [100].

## Related literature

For related structures incorporating the 1,4-bis­(4,5-dihydro-2-oxazol­yl)benzene ligand, see: Suen *et al.* (2011 ▶); Wang *et al.* (2008 ▶, 2011**a* ▶,b* ▶); Yeh *et al.* (2011 ▶).
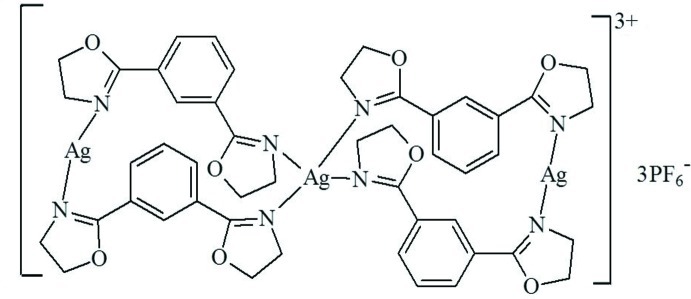


## Experimental

### 

#### Crystal data

[Ag_3_(C_12_H_12_N_2_O_2_)_4_](PF_6_)_3_
*M* *_r_* = 1623.46
Monoclinic, 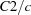

*a* = 22.7473 (16) Å
*b* = 11.4521 (19) Å
*c* = 24.1382 (15) Å
β = 116.014 (7)°
*V* = 5651.0 (11) Å^3^
*Z* = 4
Mo *K*α radiation
μ = 1.23 mm^−1^
*T* = 298 K
0.60 × 0.40 × 0.30 mm

#### Data collection

Siemens P4 four-circle diffractometer
Absorption correction: ψ scan (*XSCANS*; Siemens, 1995 ▶) *T*_min_ = 0.634, *T*_max_ = 0.964
5107 measured reflections
4976 independent reflections
3885 reflections with *I* > 2σ(*I*)
*R*_int_ = 0.020
3 standard reflections every 297 reflections intensity decay: 2.0%

#### Refinement

*R*[*F* ^2^ > 2σ(*F* ^2^)] = 0.050
*wR*(*F* ^2^) = 0.137
*S* = 1.01
4976 reflections
425 parameters
H-atom parameters constrained
Δρ_max_ = 1.02 e Å^−3^
Δρ_min_ = −0.81 e Å^−3^

### 

Data collection: *XSCANS* (Siemens, 1995 ▶); cell refinement: *XSCANS*; data reduction: *XSCANS*; program(s) used to solve structure: *SHELXS97* (Sheldrick, 2008 ▶); program(s) used to refine structure: *SHELXL97* (Sheldrick, 2008 ▶); molecular graphics: *DIAMOND* (Brandenburg, 1999 ▶); software used to prepare material for publication: *SHELXTL* (Sheldrick, 2008 ▶).

## Supplementary Material

Crystal structure: contains datablock(s) I, global. DOI: 10.1107/S1600536812034721/hy2575sup1.cif

Structure factors: contains datablock(s) I. DOI: 10.1107/S1600536812034721/hy2575Isup2.hkl

Additional supplementary materials:  crystallographic information; 3D view; checkCIF report

## Figures and Tables

**Table 1:** Selected bond lengths (Å)

Ag1—N11	2.331 (4)
Ag1—N41	2.418 (5)
Ag2—N31	2.104 (5)
Ag2—N61	2.106 (4)

**Fig. 1.**
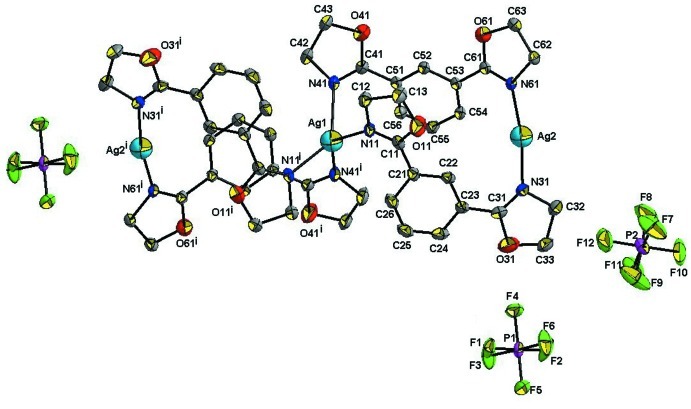
The molecular structure of the title compound. Displacement ellipsoids are drawn at the 30% probability level. H atoms have been omitted for clarity. [Symmetry code: (i) 1-x, y, 1/2-z.]

**Fig. 2.**
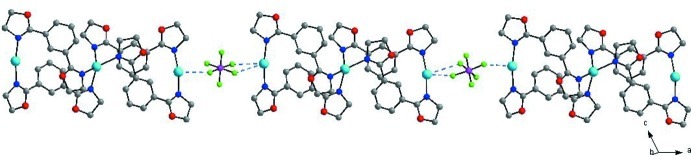
The packing diagram of the title compound, showing the one-dimensional beaded chain formed by Ag···F interactions.

## supplementary crystallographic information

### Comment

Several Ag(I), Cd(II) and Cu(II) complexes containing 1,4-bis(4,5-dihydro-2-oxazolyl)benzene ligands have been reported, which show various dimensional structures (Suen *et al.*, 2011; Wang *et al.*, 2008, 2011*a*,b; Yeh *et al.*, 2011). In the title trinuclear complex, one Ag^I^ ion, lying on a twofold axis, is coordinated by four N atoms from four 1,3-bis(4,5-dihydro-2-oxazolyl)benzene (*L*) ligands in a distorted tetrahedral geometry and the other two are each coordinated by two N atoms from two *L* ligands in a bent linear arrangement (Fig. 1, Table 1). The Ag···Ag separation in the trimer is 7.473 (1) Å. The *L* ligands show both *syn* and *anti* conformations. The PF_6_^-^ anions link the trinuclear cationic complexes *via* Ag···F interactions [2.80 (2) and 2.85 (2) Å], forming one-dimensional beaded polymeric chains along [100] (Fig. 2).

### Experimental

An aqueous solution (5.0 ml) of AgPF_6_ (3.0 mmol) was layered carefully over a methanolic solution (5.0 ml) of 1,3-bis(4,5-dihydro-2-oxazolyl)benzene (4.0 mmol) in a tube and kept it in dark. Colourless crystals were obtained after several weeks. These were washed with methanol and collected in 75.8% yield.

### Refinement

H atoms were positioned geometrically and refined as riding atoms, with C—H = 0.93 (phenyl) and 0.97 (methylene) Å and with *U*_iso_(H) = 1.2*U*_eq_(C).

### Crystal data

**Table:**

[Ag_3_(C_12_H_12_N_2_O_2_)_4_](PF_6_)_3_	*F*(000) = 3216
*M**_r_* = 1623.46	*D*_x_ = 1.908 Mg m^−^^3^
Monoclinic, *C*2/*c*	Mo *K*α radiation, λ = 0.71073 Å
Hall symbol: -C 2yc	Cell parameters from 36 reflections
*a* = 22.7473 (16) Å	θ = 4.8–12.5°
*b* = 11.4521 (19) Å	µ = 1.23 mm^−^^1^
*c* = 24.1382 (15) Å	*T* = 298 K
β = 116.014 (7)°	Plate, colourless
*V* = 5651.0 (11) Å^3^	0.60 × 0.40 × 0.30 mm
*Z* = 4	

### Data collection

**Table:**

Siemens P4 four-circle diffractometer	3885 reflections with *I* > 2σ(*I*)
Radiation source: fine-focus sealed tube	*R*_int_ = 0.020
Graphite monochromator	θ_max_ = 25.0°, θ_min_ = 2.0°
ω scans	*h* = 0→26
Absorption correction: ψ scan (*XSCANS*; Siemens, 1995)	*k* = 0→13
*T*_min_ = 0.634, *T*_max_ = 0.964	*l* = −28→25
5107 measured reflections	3 standard reflections every 297 reflections
4976 independent reflections	intensity decay: 2.0%

### Refinement

**Table:**

Refinement on *F*^2^	Primary atom site location: structure-invariant direct methods
Least-squares matrix: full	Secondary atom site location: difference Fourier map
*R*[*F*^2^ > 2σ(*F*^2^)] = 0.050	Hydrogen site location: inferred from neighbouring sites
*wR*(*F*^2^) = 0.137	H-atom parameters constrained
*S* = 1.01	*w* = 1/[σ^2^(*F*_o_^2^) + (0.0628*P*)^2^ + 34.3783*P*] where *P* = (*F*_o_^2^ + 2*F*_c_^2^)/3
4976 reflections	(Δ/σ)_max_ < 0.001
425 parameters	Δρ_max_ = 1.02 e Å^−^^3^
0 restraints	Δρ_min_ = −0.81 e Å^−^^3^

### Special details

**Table:**

Geometry. All e.s.d.'s (except the e.s.d. in the dihedral angle between two l.s. planes) are estimated using the full covariance matrix. The cell e.s.d.'s are taken into account individually in the estimation of e.s.d.'s in distances, angles and torsion angles; correlations between e.s.d.'s in cell parameters are only used when they are defined by crystal symmetry. An approximate (isotropic) treatment of cell e.s.d.'s is used for estimating e.s.d.'s involving l.s. planes.
Refinement. Refinement of *F*^2^ against ALL reflections. The weighted *R*-factor *wR* and goodness of fit *S* are based on *F*^2^, conventional *R*-factors *R* are based on *F*, with *F* set to zero for negative *F*^2^. The threshold expression of *F*^2^ > σ(*F*^2^) is used only for calculating *R*-factors(gt) *etc*. and is not relevant to the choice of reflections for refinement. *R*-factors based on *F*^2^ are statistically about twice as large as those based on *F*, and *R*- factors based on ALL data will be even larger.

### Fractional atomic coordinates and isotropic or equivalent isotropic displacement parameters (Å^2^)

**Table:**

	*x*	*y*	*z*	*U*_iso_*/*U*_eq_	Occ. (<1)
Ag1	0.5000	0.06276 (6)	0.2500	0.0564 (2)	
Ag2	0.18074 (2)	0.04964 (5)	0.26872 (2)	0.05790 (18)	
O11	0.31412 (19)	−0.1417 (4)	0.19947 (19)	0.0637 (11)	
O31	0.3068 (2)	−0.0441 (4)	0.45572 (19)	0.0675 (12)	
O41	0.3592 (2)	0.2537 (5)	0.08323 (18)	0.0694 (13)	
O61	0.14979 (18)	0.1693 (4)	0.09064 (16)	0.0532 (9)	
N11	0.4086 (2)	−0.0591 (4)	0.2133 (2)	0.0488 (11)	
N31	0.2312 (2)	0.0070 (4)	0.3628 (2)	0.0507 (11)	
N41	0.4514 (2)	0.1949 (5)	0.1635 (2)	0.0558 (12)	
N61	0.1478 (2)	0.1039 (4)	0.1765 (2)	0.0491 (11)	
C11	0.3711 (2)	−0.0898 (5)	0.2366 (3)	0.0458 (12)	
C12	0.3785 (3)	−0.0977 (6)	0.1487 (3)	0.0580 (15)	
H12A	0.4034	−0.1606	0.1423	0.070*	
H12B	0.3753	−0.0337	0.1212	0.070*	
C13	0.3111 (3)	−0.1394 (7)	0.1382 (3)	0.0712 (19)	
H13A	0.2774	−0.0860	0.1116	0.085*	
H13B	0.3021	−0.2166	0.1198	0.085*	
C21	0.3827 (3)	−0.0752 (5)	0.3012 (3)	0.0465 (12)	
C22	0.3315 (3)	−0.0577 (5)	0.3166 (2)	0.0457 (12)	
H22A	0.2888	−0.0556	0.2856	0.055*	
C23	0.3426 (3)	−0.0434 (5)	0.3773 (2)	0.0469 (12)	
C24	0.4070 (3)	−0.0470 (6)	0.4234 (3)	0.0657 (17)	
H24A	0.4155	−0.0356	0.4644	0.079*	
C25	0.4578 (3)	−0.0674 (6)	0.4080 (3)	0.0710 (19)	
H25A	0.5003	−0.0719	0.4390	0.085*	
C26	0.4465 (3)	−0.0810 (6)	0.3481 (3)	0.0639 (17)	
H26A	0.4813	−0.0942	0.3384	0.077*	
C31	0.2900 (3)	−0.0254 (5)	0.3960 (2)	0.0506 (13)	
C32	0.1977 (3)	0.0162 (6)	0.4034 (3)	0.0629 (16)	
H32A	0.1619	−0.0390	0.3911	0.075*	
H32B	0.1809	0.0944	0.4023	0.075*	
C33	0.2505 (4)	−0.0123 (8)	0.4664 (3)	0.080 (2)	
H33A	0.2603	0.0549	0.4936	0.096*	
H33B	0.2374	−0.0768	0.4846	0.096*	
C41	0.3914 (3)	0.2206 (5)	0.1427 (2)	0.0464 (12)	
C42	0.4700 (3)	0.2166 (8)	0.1130 (3)	0.081 (2)	
H42A	0.4845	0.1450	0.1013	0.098*	
H42B	0.5049	0.2738	0.1252	0.098*	
C43	0.4086 (3)	0.2623 (8)	0.0603 (3)	0.080 (2)	
H43A	0.4142	0.3426	0.0508	0.096*	
H43B	0.3968	0.2150	0.0237	0.096*	
C51	0.3518 (2)	0.2183 (5)	0.1772 (2)	0.0429 (12)	
C52	0.2861 (2)	0.1886 (5)	0.1481 (2)	0.0430 (12)	
H52A	0.2663	0.1709	0.1062	0.052*	
C53	0.2499 (2)	0.1855 (4)	0.1822 (2)	0.0407 (11)	
C54	0.2793 (3)	0.2170 (5)	0.2441 (2)	0.0469 (13)	
H54A	0.2550	0.2162	0.2667	0.056*	
C55	0.3442 (3)	0.2492 (5)	0.2721 (3)	0.0516 (14)	
H55A	0.3635	0.2712	0.3134	0.062*	
C56	0.3809 (3)	0.2492 (5)	0.2392 (3)	0.0495 (13)	
H56A	0.4249	0.2697	0.2584	0.059*	
C61	0.1804 (2)	0.1498 (5)	0.1510 (2)	0.0427 (12)	
C62	0.0795 (3)	0.0839 (7)	0.1278 (3)	0.0641 (18)	
H62A	0.0479	0.1267	0.1368	0.077*	
H62B	0.0684	0.0015	0.1238	0.077*	
C63	0.0821 (3)	0.1300 (6)	0.0701 (3)	0.0555 (14)	
H63A	0.0713	0.0690	0.0393	0.067*	
H63B	0.0518	0.1943	0.0527	0.067*	
P1	0.5000	0.4236 (2)	0.7500	0.0619 (6)	
F1	0.5316 (14)	0.3242 (15)	0.7277 (12)	0.136 (9)	0.50
F2	0.4658 (10)	0.5162 (10)	0.7741 (9)	0.102 (4)	0.50
F3	0.5624 (8)	0.503 (2)	0.7726 (12)	0.172 (9)	0.50
F4	0.4740 (10)	0.490 (2)	0.6891 (7)	0.151 (6)	0.50
F5	0.5275 (10)	0.3615 (19)	0.8141 (7)	0.135 (5)	0.50
F6	0.4354 (9)	0.3566 (19)	0.7311 (13)	0.156 (8)	0.50
P2	0.12182 (9)	0.19994 (18)	0.52246 (10)	0.0733 (5)	
F7	0.0734 (4)	0.1210 (9)	0.4738 (4)	0.231 (5)	
F8	0.0936 (4)	0.2993 (9)	0.4800 (5)	0.241 (6)	
F9	0.1540 (4)	0.1000 (7)	0.5635 (6)	0.266 (7)	
F10	0.0687 (4)	0.2167 (15)	0.5419 (5)	0.290 (8)	
F11	0.1701 (3)	0.2775 (6)	0.5752 (3)	0.156 (3)	
F12	0.1760 (4)	0.1934 (12)	0.5037 (4)	0.248 (6)	

### Atomic displacement parameters (Å^2^)

**Table:**

	*U*^11^	*U*^22^	*U*^33^	*U*^12^	*U*^13^	*U*^23^
Ag1	0.0330 (3)	0.0662 (4)	0.0635 (4)	0.000	0.0150 (3)	0.000
Ag2	0.0457 (3)	0.0879 (4)	0.0438 (3)	0.0038 (2)	0.0231 (2)	0.0128 (2)
O11	0.045 (2)	0.090 (3)	0.059 (2)	−0.027 (2)	0.0255 (19)	−0.016 (2)
O31	0.082 (3)	0.080 (3)	0.041 (2)	0.009 (2)	0.028 (2)	0.010 (2)
O41	0.056 (2)	0.114 (4)	0.048 (2)	0.017 (3)	0.031 (2)	0.018 (2)
O61	0.042 (2)	0.075 (3)	0.042 (2)	0.0003 (19)	0.0179 (16)	0.0088 (19)
N11	0.035 (2)	0.057 (3)	0.057 (3)	−0.008 (2)	0.023 (2)	−0.003 (2)
N31	0.054 (3)	0.059 (3)	0.042 (2)	−0.003 (2)	0.024 (2)	0.004 (2)
N41	0.039 (3)	0.080 (4)	0.056 (3)	0.005 (2)	0.029 (2)	0.011 (2)
N61	0.035 (2)	0.074 (3)	0.043 (2)	0.004 (2)	0.0216 (19)	0.007 (2)
C11	0.036 (3)	0.043 (3)	0.056 (3)	0.000 (2)	0.017 (2)	0.000 (2)
C12	0.048 (3)	0.067 (4)	0.063 (4)	−0.009 (3)	0.029 (3)	−0.009 (3)
C13	0.063 (4)	0.096 (5)	0.057 (4)	−0.026 (4)	0.029 (3)	−0.019 (4)
C21	0.039 (3)	0.046 (3)	0.053 (3)	−0.006 (2)	0.018 (2)	0.001 (2)
C22	0.040 (3)	0.045 (3)	0.044 (3)	−0.003 (2)	0.011 (2)	0.003 (2)
C23	0.043 (3)	0.046 (3)	0.045 (3)	−0.003 (2)	0.013 (2)	0.005 (2)
C24	0.066 (4)	0.074 (4)	0.041 (3)	−0.009 (3)	0.008 (3)	0.005 (3)
C25	0.045 (3)	0.087 (5)	0.059 (4)	−0.003 (3)	0.001 (3)	0.011 (3)
C26	0.037 (3)	0.075 (4)	0.069 (4)	−0.003 (3)	0.014 (3)	0.008 (3)
C31	0.065 (4)	0.047 (3)	0.038 (3)	−0.005 (3)	0.021 (3)	0.002 (2)
C32	0.076 (4)	0.064 (4)	0.064 (4)	0.001 (3)	0.045 (3)	0.005 (3)
C33	0.087 (5)	0.112 (6)	0.050 (4)	−0.007 (5)	0.039 (4)	−0.001 (4)
C41	0.047 (3)	0.051 (3)	0.046 (3)	0.000 (2)	0.024 (2)	0.001 (2)
C42	0.065 (4)	0.126 (7)	0.074 (4)	0.016 (4)	0.050 (4)	0.027 (4)
C43	0.069 (4)	0.128 (7)	0.061 (4)	0.005 (4)	0.046 (4)	0.011 (4)
C51	0.039 (3)	0.048 (3)	0.045 (3)	0.007 (2)	0.021 (2)	0.005 (2)
C52	0.042 (3)	0.050 (3)	0.041 (3)	0.006 (2)	0.021 (2)	0.004 (2)
C53	0.035 (3)	0.045 (3)	0.044 (3)	0.007 (2)	0.019 (2)	0.004 (2)
C54	0.045 (3)	0.059 (3)	0.045 (3)	0.010 (3)	0.027 (2)	0.005 (2)
C55	0.049 (3)	0.065 (4)	0.044 (3)	−0.001 (3)	0.023 (3)	−0.006 (3)
C56	0.039 (3)	0.060 (4)	0.050 (3)	0.003 (3)	0.020 (2)	0.000 (3)
C61	0.040 (3)	0.047 (3)	0.043 (3)	0.008 (2)	0.021 (2)	0.004 (2)
C62	0.035 (3)	0.109 (5)	0.047 (3)	−0.011 (3)	0.017 (2)	0.005 (3)
C63	0.041 (3)	0.071 (4)	0.049 (3)	0.004 (3)	0.015 (2)	0.006 (3)
P1	0.0493 (12)	0.0745 (16)	0.0784 (16)	0.000	0.0431 (12)	0.000
F1	0.21 (3)	0.091 (9)	0.20 (2)	0.038 (14)	0.18 (2)	0.011 (13)
F2	0.124 (13)	0.075 (6)	0.157 (12)	0.029 (8)	0.108 (11)	0.014 (8)
F3	0.071 (9)	0.21 (2)	0.23 (2)	−0.047 (10)	0.064 (13)	−0.034 (19)
F4	0.142 (13)	0.225 (18)	0.118 (11)	0.074 (14)	0.086 (11)	0.076 (12)
F5	0.157 (13)	0.161 (15)	0.105 (9)	0.074 (12)	0.074 (9)	0.066 (10)
F6	0.101 (11)	0.142 (19)	0.25 (3)	−0.054 (11)	0.101 (15)	−0.039 (16)
P2	0.0472 (9)	0.0794 (13)	0.0804 (12)	0.0067 (9)	0.0161 (9)	−0.0022 (10)
F7	0.147 (7)	0.200 (9)	0.208 (9)	−0.008 (6)	−0.048 (6)	−0.083 (7)
F8	0.140 (6)	0.246 (11)	0.283 (12)	0.054 (7)	0.043 (7)	0.168 (10)
F9	0.118 (6)	0.153 (7)	0.368 (14)	−0.043 (5)	−0.041 (7)	0.137 (8)
F10	0.091 (5)	0.61 (2)	0.194 (8)	0.005 (9)	0.086 (6)	−0.080 (12)
F11	0.110 (5)	0.137 (5)	0.166 (6)	−0.005 (4)	0.010 (4)	−0.052 (5)
F12	0.139 (6)	0.457 (18)	0.165 (7)	0.096 (9)	0.082 (6)	−0.042 (9)

### Geometric parameters (Å, º)

**Table:**

Ag1—N11	2.331 (4)	C32—H32A	0.9700
Ag1—N41	2.418 (5)	C32—H32B	0.9700
Ag2—N31	2.104 (5)	C33—H33A	0.9700
Ag2—N61	2.106 (4)	C33—H33B	0.9700
O11—C11	1.348 (6)	C41—C51	1.472 (7)
O11—C13	1.450 (7)	C42—C43	1.511 (10)
O31—C31	1.335 (7)	C42—H42A	0.9700
O31—C33	1.460 (8)	C42—H42B	0.9700
O41—C41	1.348 (7)	C43—H43A	0.9700
O41—C43	1.457 (7)	C43—H43B	0.9700
O61—C61	1.329 (6)	C51—C52	1.387 (7)
O61—C63	1.467 (7)	C51—C56	1.391 (7)
N11—C11	1.260 (7)	C52—C53	1.396 (7)
N11—C12	1.469 (7)	C52—H52A	0.9300
N31—C31	1.276 (8)	C53—C54	1.391 (7)
N31—C32	1.485 (7)	C53—C61	1.479 (7)
N41—C41	1.264 (7)	C54—C55	1.378 (8)
N41—C42	1.478 (7)	C54—H54A	0.9300
N61—C61	1.269 (7)	C55—C56	1.382 (7)
N61—C62	1.498 (7)	C55—H55A	0.9300
C11—C21	1.474 (8)	C56—H56A	0.9300
C12—C13	1.517 (8)	C62—C63	1.515 (8)
C12—H12A	0.9700	C62—H62A	0.9700
C12—H12B	0.9700	C62—H62B	0.9700
C13—H13A	0.9700	C63—H63A	0.9700
C13—H13B	0.9700	C63—H63B	0.9700
C21—C22	1.384 (8)	P1—F4	1.527 (12)
C21—C26	1.396 (8)	P1—F6	1.539 (16)
C22—C23	1.383 (8)	P1—F1	1.562 (13)
C22—H22A	0.9300	P1—F5	1.564 (11)
C23—C24	1.398 (8)	P1—F3	1.566 (16)
C23—C31	1.469 (8)	P1—F2	1.570 (11)
C24—C25	1.380 (10)	P2—F8	1.476 (8)
C24—H24A	0.9300	P2—F9	1.481 (7)
C25—C26	1.363 (10)	P2—F12	1.488 (8)
C25—H25A	0.9300	P2—F10	1.490 (7)
C26—H26A	0.9300	P2—F7	1.509 (7)
C32—C33	1.504 (10)	P2—F11	1.546 (6)
			
N11—Ag1—N11^i^	106.5 (2)	O61—C63—C62	104.7 (4)
N11—Ag1—N41^i^	134.17 (16)	O61—C63—H63A	110.8
N11^i^—Ag1—N41^i^	93.01 (17)	C62—C63—H63A	110.8
N11—Ag1—N41	93.01 (17)	O61—C63—H63B	110.8
N11^i^—Ag1—N41	134.17 (16)	C62—C63—H63B	110.8
N41^i^—Ag1—N41	102.5 (3)	H63A—C63—H63B	108.9
N31—Ag2—N61	169.03 (17)	F4^ii^—P1—F4	120 (2)
C11—O11—C13	105.7 (4)	F4^ii^—P1—F6	116.9 (11)
C31—O31—C33	107.0 (5)	F4—P1—F6	92.7 (16)
C41—O41—C43	105.9 (5)	F4^ii^—P1—F6^ii^	92.7 (16)
C61—O61—C63	106.7 (4)	F4—P1—F6^ii^	116.9 (11)
C11—N11—C12	107.2 (4)	F6—P1—F6^ii^	120.2 (18)
C11—N11—Ag1	131.5 (4)	F4^ii^—P1—F1^ii^	94.4 (10)
C12—N11—Ag1	120.5 (3)	F6^ii^—P1—F1^ii^	93.3 (12)
C31—N31—C32	107.7 (5)	F4^ii^—P1—F1	130.8 (17)
C31—N31—Ag2	132.2 (4)	F4—P1—F1	94.4 (10)
C32—N31—Ag2	120.1 (4)	F6—P1—F1	93.3 (12)
C41—N41—C42	106.4 (5)	F1^ii^—P1—F1	86.4 (13)
C41—N41—Ag1	119.6 (4)	F4^ii^—P1—F5^ii^	177.0 (16)
C42—N41—Ag1	129.0 (4)	F4—P1—F5^ii^	57.1 (9)
C61—N61—C62	107.9 (4)	F6—P1—F5^ii^	64.2 (9)
C61—N61—Ag2	128.5 (4)	F6^ii^—P1—F5^ii^	89.0 (10)
C62—N61—Ag2	123.4 (3)	F1^ii^—P1—F5^ii^	88.0 (11)
N11—C11—O11	118.0 (5)	F1—P1—F5^ii^	51.1 (9)
N11—C11—C21	126.8 (5)	F4^ii^—P1—F5	57.1 (9)
O11—C11—C21	115.2 (5)	F4—P1—F5	177.0 (17)
N11—C12—C13	103.9 (5)	F6—P1—F5	89.0 (10)
N11—C12—H12A	111.0	F6^ii^—P1—F5	64.2 (9)
C13—C12—H12A	111.0	F1^ii^—P1—F5	51.1 (9)
N11—C12—H12B	111.0	F1—P1—F5	88.0 (11)
C13—C12—H12B	111.0	F5^ii^—P1—F5	125.9 (18)
H12A—C12—H12B	109.0	F4^ii^—P1—F3	57.7 (9)
O11—C13—C12	104.0 (5)	F4—P1—F3	87.4 (11)
O11—C13—H13A	111.0	F6—P1—F3	173.3 (12)
C12—C13—H13A	111.0	F6^ii^—P1—F3	65.4 (11)
O11—C13—H13B	111.0	F1^ii^—P1—F3	141.7 (17)
C12—C13—H13B	111.0	F1—P1—F3	93.3 (12)
H13A—C13—H13B	109.0	F5^ii^—P1—F3	121.0 (10)
C22—C21—C26	119.2 (6)	F5—P1—F3	90.6 (16)
C22—C21—C11	121.3 (5)	F4^ii^—P1—F3^ii^	87.4 (11)
C26—C21—C11	119.5 (5)	F4—P1—F3^ii^	57.7 (9)
C23—C22—C21	121.1 (5)	F6—P1—F3^ii^	65.4 (11)
C23—C22—H22A	119.4	F6^ii^—P1—F3^ii^	173.3 (12)
C21—C22—H22A	119.4	F1^ii^—P1—F3^ii^	93.3 (12)
C22—C23—C24	118.8 (6)	F1—P1—F3^ii^	141.7 (17)
C22—C23—C31	123.2 (5)	F5^ii^—P1—F3^ii^	90.6 (16)
C24—C23—C31	118.0 (5)	F5—P1—F3^ii^	121.0 (10)
C25—C24—C23	119.9 (6)	F3—P1—F3^ii^	109.4 (18)
C25—C24—H24A	120.0	F4^ii^—P1—F2	49.6 (8)
C23—C24—H24A	120.0	F4—P1—F2	88.4 (9)
C26—C25—C24	121.0 (6)	F6—P1—F2	83.2 (11)
C26—C25—H25A	119.5	F6^ii^—P1—F2	142.3 (15)
C24—C25—H25A	119.5	F1^ii^—P1—F2	89.3 (8)
C25—C26—C21	119.9 (6)	F1—P1—F2	175.7 (8)
C25—C26—H26A	120.0	F5^ii^—P1—F2	128.7 (14)
C21—C26—H26A	120.0	F5—P1—F2	89.3 (8)
N31—C31—O31	116.8 (5)	F3—P1—F2	90.1 (9)
N31—C31—C23	128.2 (5)	F4^ii^—P1—F2^ii^	88.4 (9)
O31—C31—C23	115.0 (5)	F4—P1—F2^ii^	49.6 (8)
N31—C32—C33	103.9 (5)	F6—P1—F2^ii^	142.3 (15)
N31—C32—H32A	111.0	F6^ii^—P1—F2^ii^	83.2 (11)
C33—C32—H32A	111.0	F1^ii^—P1—F2^ii^	175.7 (8)
N31—C32—H32B	111.0	F1—P1—F2^ii^	89.3 (8)
C33—C32—H32B	111.0	F5^ii^—P1—F2^ii^	89.3 (8)
H32A—C32—H32B	109.0	F5—P1—F2^ii^	128.7 (14)
O31—C33—C32	104.3 (5)	F3^ii^—P1—F2^ii^	90.1 (9)
O31—C33—H33A	110.9	F2—P1—F2^ii^	95.1 (9)
C32—C33—H33A	110.9	F6^ii^—F1—F5^ii^	133 (2)
O31—C33—H33B	110.9	F6^ii^—F1—P1	69.6 (14)
C32—C33—H33B	110.9	F5^ii^—F1—P1	64.5 (9)
H33A—C33—H33B	108.9	F3^ii^—F2—F4^ii^	132.4 (16)
N41—C41—O41	118.5 (5)	F3^ii^—F2—P1	70.6 (13)
N41—C41—C51	126.3 (5)	F4^ii^—F2—P1	63.5 (8)
O41—C41—C51	115.2 (5)	F2^ii^—F3—F4^ii^	116.2 (18)
N41—C42—C43	105.0 (5)	F2^ii^—F3—P1	70.9 (11)
N41—C42—H42A	110.8	F4^ii^—F3—P1	59.8 (8)
C43—C42—H42A	110.8	F2^ii^—F3—F6^ii^	96 (2)
N41—C42—H42B	110.8	F4^ii^—F3—F6^ii^	89 (2)
C43—C42—H42B	110.8	P1—F3—F6^ii^	56.5 (9)
H42A—C42—H42B	108.8	F2^ii^—F4—F5^ii^	104.8 (12)
O41—C43—C42	103.9 (5)	F2^ii^—F4—F3^ii^	105.1 (17)
O41—C43—H43A	111.0	F5^ii^—F4—F3^ii^	97.0 (18)
C42—C43—H43A	111.0	F2^ii^—F4—P1	66.9 (8)
O41—C43—H43B	111.0	F5^ii^—F4—P1	62.7 (7)
C42—C43—H43B	111.0	F3^ii^—F4—P1	62.5 (8)
H43A—C43—H43B	109.0	F1^ii^—F5—F4^ii^	106.5 (13)
C52—C51—C56	120.5 (5)	F1^ii^—F5—P1	64.4 (8)
C52—C51—C41	120.6 (5)	F4^ii^—F5—P1	60.2 (7)
C56—C51—C41	118.9 (5)	F1^ii^—F5—F6^ii^	97.1 (15)
C51—C52—C53	119.3 (5)	F4^ii^—F5—F6^ii^	90.2 (15)
C51—C52—H52A	120.3	P1—F5—F6^ii^	57.2 (7)
C53—C52—H52A	120.3	F1^ii^—F6—P1	72.1 (15)
C54—C53—C52	119.8 (5)	F1^ii^—F6—F5^ii^	107 (2)
C54—C53—C61	121.4 (4)	P1—F6—F5^ii^	58.6 (7)
C52—C53—C61	118.8 (5)	F1^ii^—F6—F3^ii^	113 (3)
C55—C54—C53	120.3 (5)	P1—F6—F3^ii^	58.1 (8)
C55—C54—H54A	119.9	F5^ii^—F6—F3^ii^	84.0 (17)
C53—C54—H54A	119.9	F8—P2—F9	175.8 (7)
C54—C55—C56	120.3 (5)	F8—P2—F12	89.7 (6)
C54—C55—H55A	119.8	F9—P2—F12	86.1 (7)
C56—C55—H55A	119.8	F8—P2—F10	87.3 (7)
C55—C56—C51	119.7 (5)	F9—P2—F10	96.8 (8)
C55—C56—H56A	120.1	F12—P2—F10	175.4 (9)
C51—C56—H56A	120.1	F8—P2—F7	88.6 (6)
N61—C61—O61	117.7 (5)	F9—P2—F7	92.0 (6)
N61—C61—C53	126.2 (5)	F12—P2—F7	98.8 (6)
O61—C61—C53	116.1 (4)	F10—P2—F7	84.6 (6)
N61—C62—C63	103.0 (4)	F8—P2—F11	93.7 (6)
N61—C62—H62A	111.2	F9—P2—F11	85.9 (5)
C63—C62—H62A	111.2	F12—P2—F11	84.0 (5)
N61—C62—H62B	111.2	F10—P2—F11	92.8 (6)
C63—C62—H62B	111.2	F7—P2—F11	176.4 (6)
H62A—C62—H62B	109.1		

Symmetry codes: (i) −*x*+1, *y*, −*z*+1/2; (ii) −*x*+1, *y*, −*z*+3/2.
